# Histone Modification on Parathyroid Tumors: A Review of Epigenetics

**DOI:** 10.3390/ijms23105378

**Published:** 2022-05-11

**Authors:** Luiz C. Conti de Freitas, Rogerio M. Castilho, Cristiane H. Squarize

**Affiliations:** 1 Department of Ophthalmology, Otolaryngology and Head and Neck Surgery, Ribeirao Preto Medical School, University of São Paulo, Ribeirao Preto 14049-900, SP, Brazil; lcconti@fmrp.usp.br; 2 Laboratory of Epithelial Biology, Department of Periodontics and Oral Medicine, University of Michigan School of Dentistry, Ann Arbor, MI 48109-1078, USA; rcastilh@umich.edu

**Keywords:** parathyroid adenoma, epigenetics, hyperparathyroidism, histone

## Abstract

Parathyroid tumors are very prevalent conditions among endocrine tumors, being the second most common behind thyroid tumors. Secondary hyperplasia can occur beyond benign and malignant neoplasia in parathyroid glands. Adenomas are the leading cause of hyperparathyroidism, while carcinomas represent less than 1% of the cases. Tumor suppressor gene mutations such as *MEN1* and *CDC73* were demonstrated to be involved in tumor development in both familiar and sporadic types; however, the epigenetic features of the parathyroid tumors are still a little-explored subject. We present a review of epigenetic mechanisms related to parathyroid tumors, emphasizing advances in histone modification and its perspective of becoming a promising area in parathyroid tumor research.

## 1. Introduction

The parathyroid glands were first identified in an Indian rhinoceros in 1849 by Sir Richard Owen [1] and later described in various animals and humans by Ivar Sandström [2], who named them *parathyroids*. The parathyroids usually present as four tiny glands located close to the superior and inferior poles of the thyroid gland [3,4,5,6,7,8]. Parathyroid hormone (PTH) plays an essential role in calcium serum-level regulation. Even a slight reduction or elevation of calcium levels can stimulate or suppress parathyroid hormone secretion [9]. Parathyroid disorders are among the highest incidence of endocrine diseases, being the third most frequent, only behind thyroid disease and diabetes. Parathyroid tumors are the second most common endocrine tumors, just behind thyroid tumors [10].

The most relevant diseases that affect the parathyroid glands include clinical conditions with elevated serum PTH levels, such as primary hyperparathyroidism (pHPT) and secondary hyperparathyroidism (sHPT). The population incidence of pHPT from 1995 to 2010 was 65.5 per 100,000 women and 24.7 per 100,000 men [11], while the prevalence of sHPT in patients with renal disease ranged between 20% and 80% [12,13]. pHPT is an intrinsic gland disorder with elevation in hormone secretion, and sHPT is an extrinsic disorder, usually caused by renal failure or vitamin D insufficiency [14], which stimulates parathyroid secretion and gland enlargement. Additionally, pHPT can also be caused by a single adenoma, multiple parathyroid disease, or carcinoma of the parathyroid. Approximately 80% to 85% of pHPT is caused by a single adenoma in one of the four parathyroid glands [15,16], while the remaining cases involve all four parathyroid glands, which may be sporadic or familial. Familial syndromes are mainly multiple endocrine neoplasms (MEN) types 1 and 2A, hyperparathyroidism-tumor of the mandible, and isolated familial hyperparathyroidism. Although malignant parathyroid lesions are uncommon, they often lead to severe hypercalcemia and disturbing clinical symptoms, such as fatigue, weakness, weight loss, anorexia, and bone pain [17].

The role of genetic alterations in parathyroid tumors has been extensively studied [14,18,19,20,21,22,23]. Both oncogenes and tumor suppressor genes, such as *MEN1* and *CDC73* (previously named *HRPT2*), are involved in parathyroid disease pathogenesis. *MEN1*, a tumor suppressor gene, was first studied as a germinative mutation associated with multiple endocrine neoplasia type 1. Its somatic mutations are known to be the most frequent mutations identified in parathyroid tumors [24,25]. The overexpression of the oncogene *cyclin D1/PRAD1* is observed in 20% to 40% of parathyroid adenomas, and it is considered a molecular driver of sporadic adenomas [18,26]. As *Cyclin D1* has a vital role in cell cycle regulation, other molecules that affect its function could be associated with tumorigenesis. Thus, there is some evidence that cyclin-dependent kinase inhibitors (*CDKI*) gene mutations can lead to parathyroid tumor development, particularly the mutations in *CDKN1B/p27* gene [27]. Other genes presenting somatic mutations are *β-catenin*, *POT1*, and *EZH2* [23,28].

Mutations on *CDC73*, *MEN1*, *HIC1*, *EZH2*, and *β-catenin* genes, demonstrated to be involved in parathyroid tumors, seem to implicate epigenetic modifications in their mechanism of tumorigenesis. The epigenetic modifications are mechanisms that control gene activation and silencing and are involved in disease development [29,30,31]. They are related to (1) DNA methylation, (2) histone modifications, and (3) chromatin remodeling. DNA methylation consists of covalent binding of the methyl group at cytosine (position 5′) to guanine, promoted by DNA methyltransferase 1 (DNMT1). Methylation promotes the silencing of gene transcription and is a factor involved in developing autoimmune neurodegenerative diseases and cancer [32]. Histones are protein components of chromatin. The activation of an N-terminal group susceptible to covalent binding may lead to modification in DNA stability that affects transcription. Chromatin remodeling is mainly affected by nucleosome positioning around the transcription start site, so nucleosome position is also linked to DNA transcription [33,34]. Different epigenetic mechanisms can interfere with each of the other functions and are also controlled by regulatory molecules such as histone variants, noncoding RNA, and others. The final effect over DNA transcription is the sum of the interaction of these different mechanisms [32].

Histones are proteins that regulate the DNA condensation mechanism. The DNA strand completes two turns around the histone to form the nucleosome, stabilizing the chromatin. Each histone is composed of two copies of the H2A/H2B dimers and one H3/H4 tetramer and has an N-terminal group that is susceptible to covalent bonds, especially from methyl (methylation) and acetyl (acetylation) groups, which can lead to variations in the DNA stability. Other reactions, such as phosphorylation, ubiquitination, biotinylation, sumoylation, and proline isomerization, are likely to occur, leading to histone modification. The imbalance between histone acetylation and deacetylation may favor the development of tumors [35]. The histone acetylation promotes DNA decondensation, “opening” the DNA and favoring the transcription of several genes.

## 2. Epigenetics of the Parathyroid Glands

Since most malignant lesions have presented DNA hypomethylation, studies were carried out to set up the parathyroid methylation profile. Global methylation of parathyroid tumors did not show differences compared to normal tissues [36]. However, the effect of DNA methylation on silencing tumor suppressor genes has been demonstrated to be associated with the development of parathyroid tumors [37,38]. It was observed that about 18% of the parathyroid carcinomas showed specific methylation of the *CDC73* gene. The same study evaluated 37 sporadic adenomas, and none has shown methylation changes compared to normal tissue [39]. Other specific genes whose pathways are linked to the development of parathyroid tumors, such as *RIZ1*, *APC*, *RASSF1A*, *CDKN2A/p16*, *CDKN2B/p15*, *RB1*, *WT1*, *GATA4*, *PYCARD*, *SFRP1* and *SFRP2*, also appeared to be hypermethylated in both malignant and benign parathyroid tumors [40]. In contrast to tumorigenesis-related gene methylation, parathyroid adenomas did not show methylation of genes coding calcium or vitamin D receptors [41], which are inhibited in this condition [42]. The same results were observed in parathyroid hyperplasia caused by secondary hyperparathyroidism to chronic renal disease. Therefore, although reduced sensitivity to calcium and vitamin D was detected [9] in hyperparathyroidism, the methylation does not seem to explain the low expression of genes coding for calcium or vitamin D receptors.

Another mechanism of gene expression regulation is the control by microRNAs (miRNAs), short noncoding RNA segments that inhibit the translation or degrading of mRNAs [43,44]. In vitro studies have shown that appropriate miRNA synthesis and maturation are essential for PTH secretion by the gland in response to hypocalcemia [45]. The miRNA let-7 family is the most expressed in normal parathyroids, followed by the miR-30 and miR-141/miR200 families [46]. Additionally, the blockade of miRNA let-7 in an in vitro study led to increased PTH secretion by the parathyroid gland [45,47]. Moreover, an animal model of secondary hyperparathyroidism demonstrated an increased miR-21, miR-29, miR30, miR-141, and miR-148, and reduced expression of miR10, miR-25, and miR-125 families [45]. The miRNA expression seems to be inhibited in parathyroid carcinomas. A more significant change was observed in miRNA-126, which showed reduced expression in carcinomas compared to benign parathyroid disease [48]. The miRNA-126 appears to be related to the migration and angiogenesis process, and its deregulation has been previously associated with other malignant neoplasia [49,50]. Recent studies have been conducted to determine an association between long-chain noncoding RNA (lncRNA) and neoplasia development [51,52,53]; lncRNA is a chain of more than 200 nucleotides with no protein-coding function but with activity in regulating gene expression. Zhang et al. (2019) observed that six *CDC73*-mutant parathyroid carcinoma samples showed a significantly higher expression of PVT1 (plasmacytoma variant translocation 1) and lower expression of *GLIS2-AS1* (GLIS2 antisense RNA 1) compared to parathyroid carcinoma samples without *CDC73* mutation, suggesting an association of changes in lncRNAs with modification of the *CDC73* gene [52].

Parafibromin protein translated from *CDC73* gene [54] binds to RNA polymerase II as part of *PAF1,* a transcriptional regulatory complex; however, the mechanism by which parafibromin loss function can lead to neoplastic transformation is not entirely understood. As a protein associated with the unphosphorylated form of RNA polymerase II, it is involved in the regulation of translation, including histone mRNA [54,55,56]. In addition, the loss of expression of *PAF1* components also affects the ubiquitination and methylation of H3 histones [55]. Furthermore, there is a modulation of H1.2, H2A, H2B histones, which are suppressed in the presence of mutation of the *CDC73* gene, thus suggesting that parafibromin may be a crucial chromatin-modifying factor [57]. In addition, the Menin protein transcribed from the *MEN1* gene and strongly correlated to multiple endocrine neoplasia type I has shown an important role in epigenetic regulation based on the modulation of DNA methylation of specific genes such as the cyclin-dependent kinase inhibitor 2A (*CDKN2A*), Ras association domain family member 1 (*RASSF1A*), and adenomatous polyposis coli (*APC*) [36,58]. However, it plays a fundamental role in the *MLL2*, a methyltransferase complex linked to histone methylation [59,60,61]. It is described as a tumor suppressor and a member of the *SET* family of proteins with histone 3 lysine 4 methyltransferase activity that was found to be mutated in cancer types. Therefore, changes happened in the tumor suppressor genes *MEN1* and *CDC73*, triggering disequilibrium of H3K4 and H3K9 activities [62,63,64].

**Figure 1:**
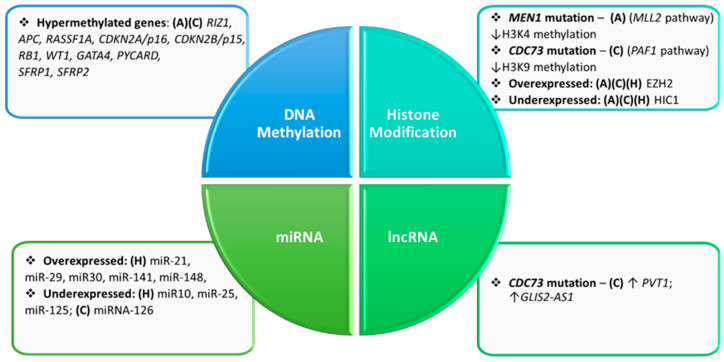
Diagram showing critical molecules related to epigenetic features of adenomas (A), carcinoma (C), and hyperplasia (H) of parathyroid glands. The epigenetics data are presented according to mechanism modality (DNA methylation, histone modification, microRNA (miRNA), and long noncoding RNA (lncRNA)) associated with the most relevant findings related to parathyroid tumorigenesis.

Lastly, *HIC1*, *EZH2* and *β-catenin* seem to present epigenetics implications on parathyroid tumorigenesis. The *HIC1* (hypermethylated gene in cancer 1) gene is a tumor suppressor and a regulator of cell growth. The low expression of the *HIC1*, observed in hyperplasia [65], benign and malignant neoplasms of the parathyroid glands [66,67], and other tumor types [68,69], has been associated with histones H3K27 methylation [70]. The *EZH2* gene (enhancer of Zeste 2 polycomb repressive complex 2 subunit) codes for a member of the polycomb-group (PcG) family. These are proteins involved in maintaining the repressive transcriptional status and lysine methylation in histones is one of their mechanisms. *EZH2* is overexpressed in parathyroid tumors regardless of their malignant or benign origin [71]. Since post-translational histone modification has been studied based on the methylation process [72,73,74], no studies were conducted on histone acetylation in parathyroid tumors (Figure 1).

Recent studies have addressed epigenetic features in not just the chemical changes of DNA or histone but suggest that conformational DNA architecture could interfere in genome function, attempting to characterize the three-dimensional DNA organization and its impact on gene expression [75,76,77]. Although histones are recognized as a crucial factor in gene expression regulation, it is not clear how histone modification leads to a conformational change in chromatin organization. Computational models have been suggested as a tool to simulate chromatin structure under the effect of different variables, including histone status [78]. In colorectal cancer, the chromatin structure seems more homogeneous in 3D space; however, different methylation patterns were observed according to the compartment analyzed. It was demonstrated that H3K27me3 has a vital role in this process [79].

Cancer stem cells are another target in the epigenetics of cancer research. These cells, present in neoplasia, began to be considered responsible for tumor aggressiveness, contributing to the local progression and metastatic dissemination. In parathyroid adenomas, stem cell population represents 10.97% of all cells, confirming that it is also present in benign diseases [80]. The Wnt/β-catenin pathway is involved in the differentiation of cancer stem cells through changes in DNA methylation that lead to the silencing of various Wnt inhibitors and acetylation of H3K16, which may contribute to tumor progression [81,82]. Thus far, no studies have been conducted to describe these cells’ epigenetic characteristics in parathyroid adenomas or carcinomas.

## 3. Potential of Histone Manipulation in Therapeutics

Epigenetic characteristics have been studied in several types of tumors, including those of the head and neck region, to clarify the mechanism of tumor progression [83,84,85,86,87]. Global DNA hypomethylation has been suggested to predict the prognosis of oropharyngeal squamous cell carcinoma, with 3.5 times higher risk of early recurrence [87,88]. On the other hand, as shown in parathyroid, hypermethylation of specific genes such as *p16* [89], *PTEN* [90], *DAPK* [91], and *RASSF1* [92] has been pointed out as a possible silencing mechanism of tumor suppressor genes, promoting the development of neoplasia.

Histone acetylation leads to decondensation with the “opening” of DNA, favoring the transcription of various genes [35]. Histone hyperacetylation can be triggered by the overexpression of inhibitors of histone deacetylation (HDAC) enzymes [93,94]. Some drugs interfering with histone acetylation have been developed, acting as specific or multiple class HDAC inhibitors and causing overexpression of p21, a cyclin-dependent kinase, interruption of the cell cycle at G2/M, and cell death in head and neck cancer cell lines [95]. Prystowsky et al. (2009) [93] observed that LBH589, an HDAC inhibitor, is associated with the interruption of the cell cycle at the G2/M phase in a cell line of pharyngeal cancer. Koike et al. (2017) [94] observed that using an HDAC inhibitor in head and neck cancer cells led to increased expression of dermatopontin, a protein regulating tumor dissemination. The induction of acetylation with an HDAC inhibitor also reduced the proliferation of cancer stem cells in tongue squamous cell carcinoma, although paradoxically increasing the expression of *BMI-1*, an oncogene associated with tumor aggressiveness [96]. A previous study demonstrated that H3 histones are hypoacetylated in head and neck squamous cells. We analyzed the expression of acetyl-histone H3 at lys9 (H3K9ac) in oral cancer and observed that histone H3 hypoacetylation was correlated with a worse prognosis for these tumors [97]. We also detected stem cells in cystic adenoid carcinoma lines of the salivary gland effectively reduced after treatment with Vorinostat; however, the combination of Vorinostat and cisplatin was highly effective in depleting stem cells of cancer and reducing tumor viability, suggesting an epigenetic remodeling [83,98].

Studies of neoplastic tissue of the parathyroid gland have focused on describing the profile of DNA and histone methylation. It has been poorly studied in histone acetylation patterns, even though this pattern was altered in other tissues of the head and neck region [61,99,100]. The thyroid gland develops tumors with high histone acetylation [101]. On the other hand, preliminary studies on prostate [102,103] and neuroendocrine tumors from the lung [104] have demonstrated histone acetylation reduction with increased tumor aggressiveness.

The association of post-translational histone modification mechanisms with tumorigenesis has opened new possibilities for identifying cancer therapy targets. The first observed was the reduction in lysine 16 acetylation and lysine 20 methylation in the H4 histone of neoplastic cells [105], showing that these could be possible targets of epigenetic modification. Pharmacological action on histone acetylation has played an outstanding role in this field. HDACs are essential for gene transcriptional activity, and a change in their expression has been demonstrated in tumors, leading to the rapid development of inhibitors of this enzyme activity recommended for their antitumor activity. The US FDA has approved Vorinostat, Romidepsin, Belinostat, and Panobinostat for use in various malignant neoplasm [106]. No studies have been conducted thus far to test the application of HDAC inhibitors to malignant or benign neoplasia of the parathyroid glands, nor has the global acetylation profile been demonstrated in different diseases affecting these glands.

Studies have been conducted to test the application of histone acetylation to the prognosis [97,107,108,109,110] or differential diagnosis [111] of benign and malignant neoplasia. Additionally, the high histone deacetylation in neoplasia has motivated studies to evaluate the capacity of HDAC histone deacetylation inhibitors to prevent cancer development. Sulforaphane, which has HDAC inhibitory activity, led to increased acetylation in colon and prostate cancer with reduced p21 and BAX, leading to the interruption of the cell cycle or apoptosis, and thus preventing cancer [112,113,114]. Formulations for the topical application of HDAC MS-275 have been tested in skin squamous cell carcinomas induced by UVB in rats, appearing to be promising for chemopreventive use [115]. Clinical situations known to lead to hyperparathyroidism are still waiting for an agent that might prevent the disease. Thus far, the application of HDAC inhibitors to parathyroid pathologies diseases has not been studied systematically.

## 4. Conclusions

In conclusion, epigenetic changes have the potential to demonstrate a relevant role in parathyroid gland tumors. The association between mechanisms of histone modification and tumorigenesis has opened a new research possibility in identifying diagnostic markers and therapeutic targets. The knowledge generated from histone studies can potentially be applied to differentiate benign and malignant parathyroid tumors. In secondary hyperparathyroidism, it will help to understand the pathophysiology and progression features of the disease. Additionally, the data obtained can determine whether parathyroid diseases are potentially susceptible to treatments targeting the histone profile.

Although many advances were observed in histone acetylation and its involvement in tumors, it was insufficiently considered in parathyroid tumors. Clarifying mechanisms and testing agents that interfere in histone acetylation can become a promising area of research in parathyroid diseases with potential application in diagnosing, preventing, and treating parathyroid tumors. The epigenetic feature of the parathyroid is an entirely new and unexplored area of investigation. Several points remain to be addressed, mainly on the role of histone.

## Data Availability

Not applicable.
